# An estimate is worth about a thousand experiments: using order-of-magnitude estimates to identify cellular engineering targets

**DOI:** 10.1186/s12934-018-0979-7

**Published:** 2018-08-30

**Authors:** Kevin James Metcalf, Marilyn F. Slininger Lee, Christopher Matthew Jakobson, Danielle Tullman-Ercek

**Affiliations:** 1 Department of Chemical and Biomolecular Engineering, University of California, Berkeley, CA 94720 USA; 2 Department of Chemical and Biological Engineering, Northwestern University, Evanston, IL 60208 USA; 3 Present Address: Department of Biomedical Engineering, Northwestern University, Evanston, IL 60208 USA; 4 Present Address: U.S. Army Edgewood Chemical Biological Center, Gunpowder, MD 21010 USA; 5 Present Address: Department of Chemical and Systems Biology, Stanford University School of Medicine, Stanford, CA 94305 USA

**Keywords:** Bioprocess, Synthetic biology, Metabolic engineering

## Abstract

Biotechnological processes use microbes to convert abundant molecules, such as glucose, into high-value products, such as pharmaceuticals, commodity and fine chemicals, and energy. However, from the outset of the development of a new bioprocess, it is difficult to determine the feasibility, expected yields, and targets for engineering. In this review, we describe a methodology that uses rough estimates to assess the feasibility of a process, approximate the expected product titer of a biological system, and identify variables to manipulate in order to achieve the desired performance. This methodology uses estimates from literature and biological intuition, and can be applied in the early stages of a project to help plan future engineering. We highlight recent literature examples, as well as two case studies from our own work, to demonstrate the use and power of rough estimates. Describing and predicting biological function using estimates guides the research and development phase of new bioprocesses and is a useful first step to understand and build a new microbial factory.

## Background

**Fig. 1:**
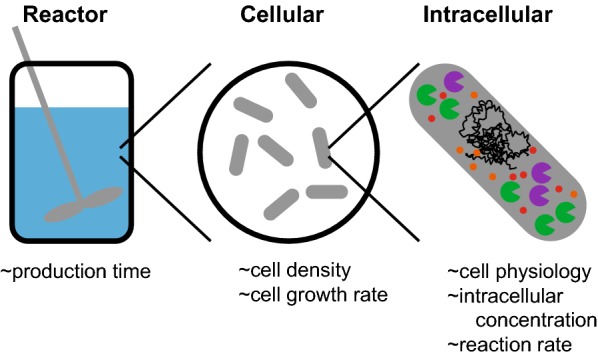
Order-of-magnitude estimates are made across different scales of a bioprocess

Every microbial production process begins with an exciting but daunting set of problems—we want to know if the process is feasible, and how to increase production of a complex biological system or pathway when the best approach may be non-obvious. These problems are particularly difficult if the system or pathway in question has never been used in an engineering context before. In the following review, we outline the methodology our group developed to inform these decisions. We combine simple biological and biochemical observations [1] with intuitive estimates to identify the aspects of the biological system that will have the greatest impact on product yield to guide engineering efforts (Fig. 1). Many approximate values come from the BioNumbers database [2], and we cite the BioNumber identification number (BNID), where applicable. We also use significant figures in the estimates to signify the degree of precision in an estimate, following rules outlined in the text *Cell Biology by the Numbers* [3]. Simultaneously, we identify other aspects of a system that are unlikely to significantly impact our targets, in order to exclude these from our engineering efforts. This exercise is a useful addition to the planning stages of any biological engineering endeavor. Below, we discuss examples from the literature, and we use case studies from our work to outline the molecular-level estimates of achievable cellular behaviors. At the end, we describe a decision scheme for applying rough estimates to a new bioprocess. We encourage others in the field to include their rough estimates for process feasibility and engineering targets in their published work.

## Estimates for production of artemisinin

Rough estimates help to characterize complex biological systems and improve product yield. In a landmark study, Keasling and coworkers used *Saccharomyces cerevisiae* to produce artemisinic acid, a precursor to the antimalarial drug artemisinin [4]. After an economic analysis of the process, they targeted a titer of 25 g/L in production, a feat they recently achieved [5]. We show a rough estimate to justify, in retrospect, this effort. The enzyme amorphadiene synthase (ADS) from *Artemisia annua* catalyzes the cyclization of farnesyl diphosphate to amorpha-4,11-diene a turnover number of 0.2 s^−1^ [6], which is relatively slow compared to the other enzymes in the pathway [7] (Table 1).

**Table 1:** Turnover number (*k*_*cat*_) for enzymes with published kinetic data used in the artemisinic acid pathway in *S. cerevisiae* [8]

Enzyme	*k*_*cat*_ (s^−1^)	Reference
ERG10	NR	
ERG13	NR	
tHMG1	0.4^a^	[9]
ERG12	16	[10]
ERG8	40^a^	[11]
MVD1	5	[12]
IDI1	7^a^	[13]
ERG20	NR	
ADS	0.2	[6]
CYP71AV1/CPR1/CYB5	NR	
ADH1	41	[5]
ALDH1	1.5	[14]

*NR* not reported

^a^Estimated from specific activity

We therefore assume that this enzyme is substrate-saturated and catalyzes the rate-determining reaction step. Using BioNumbers estimates for *S. cerevisiae*, as well as experimental values from their recent publication [5], we estimate that this process can yield up to 16 g/L artemisinic acid (calculation 1).

### Estimated maximum titer of artemisinic acid in *S. cerevisiae* (BNIDs 104150 and 100986)

1 $$\begin{aligned}\left( {1.3} \times 10^{6} \frac{\text{ ADS}}{\text{cell}}\right)\left(\frac{1 \text{ mole ADS}}{{6.02} \times 10^{23} \text{ molecules ADS}} \right)\\ & \times ({15} \text{ OD}) \left( \frac{3 \times 10^7 \text{ cells}}{\text{OD} \times \text{mL}} \right) \left( 0.2 \text{ s}^{-1} \right) \left( \frac{60^2 \text{ s}}{1 \text{ h}} \right)\\ & \times (100 \text{ h})\left( \frac{10^3 \text{ mL}}{1 \text{ L}} \right) \left( \frac{234 \text{ g}}{\text{mol}} \right) \approx {16} \frac{\text{ g}}{\text{L}} \end{aligned}$$

This estimate assumes that ADS has an abundance of 1.3 × 10^6^ enzymes per cell, which is the upper limit of native protein copies in a yeast cell (BNID 104150). This expression level is ~ 3% of the total protein (BNID 110550), and overexpression to 2 × 10^6^ ADS/cell would result in the target titer of 25 g/L. This analysis gives us an intuition for the bioprocess and serves as a benchmark for the physical limitations of this cellular process. Further increases to the product titer could be achieved by increasing the cell density, culture time, and reaction rate of ADS. A limitation of the analysis presented here is that the rate-determining enzyme must be known, and its kinetic parameters measured or estimated. When faced with a poorly defined set of enzymes, there is no substitute for intuition and accurate guesses of enzyme activity. To aid in assessing processes with limited kinetic information a lower bound for *k*_*cat*_ of 0.1 s^−1^ captures the large majority of known activities [15].

## Estimates for production of electrical energy

This type of analysis is not limited to the production of molecules—microbial production of high-energy electrons is also amenable to analysis using rough estimates. For example, in a microbial fuel cell, bacteria are used as a catalyst to convert carbon-based chemical energy to electrical energy. Chaudhuri and Lovley [16] showed that the rate of metabolism, efficiency of electron transfer, and microbial density on the electrode are determining factors for predicting the current density of a microbial fuel cell. In order to improve fuel cell performance, which parameter should an engineer first modify? The authors used a straightforward calculation to determine that electron transfer efficiency is already quite efficient—the bacterium *Rhodoferax ferrireducens* achieved a yield of 740 coulombs (C) of the theoretical limit of 900 C that can be extracted by the complete oxidation of the 0.39 mmol of glucose fed. On the other hand, they found that current density is improved by selecting appropriate electrode materials. Interestingly, the authors observed a twofold increase in current density with a new anode material, which correlated with a twofold increase in microbial density on the electrode. Using the authors’ measurement of 0.086 mg protein/cm^2^, we estimate this new material resulted in 6 × 10^8^ cells/cm^2^ cell density on the anode (calculation 2).

### Experimental cell density on anode (BNIDs 109352 and 103904)

2 $$\left( {\frac{{0.086\,{\text{mg}}\,{\text{protein}}}}{{{\text{cm}}^{ 2} }}} \right)\left( {\frac{{2\,{\text{mg}}\,{\text{DCW}}}}{\text{mg protein}}} \right)\left( {\frac{\text{cell}}{{ 2 8 0 {\text{ fg}}}}} \right)\left( {\frac{{10^{12} \,{\text{fg}}}}{{ 1 {\text{ mg}}}}} \right) \approx 6\, \times \,10^{8} \,\frac{{\text{cells}}}{{{{\text{cm}}}^{ 2} }}$$

Assuming that ~ 1/6 of the 6 μm^2^ bacterial surface area (BNID 101792) is in contact with the electrode, we estimate up to 1 × 10^8^ cells/cm^2^ could be attached to the anode (calculation 3).

### Maximum cell density on anode

3 $$\left( {\frac{\text{cell}}{{1\,\upmu{\text{m}}^{2} }}} \right)\left( {\frac{{\left( {10^{6} } \right)^{2} {\mkern 1mu}\upmu{\text{m}}^{2} }}{{\left( {10^{2} } \right)^{2} {\mkern 1mu} {\text{cm}}^{2} }}} \right) \approx 1 \times \,10^{8} {\mkern 1mu} \frac{{\text{cells}}}{{{{\text{cm}}}^{2} }}$$

This theoretical estimate is within one order of magnitude of the experimental estimate and suggests that the current density cannot be improved by increasing microbial density on the anode, as the anode surface is likely already saturated with bacteria. Further improvements to this system may also focus on the rate of metabolism of the bacterial cell. We support this claim using values estimated for *Escherichia coli*, which is similar in size and shape to *R. ferrireducens* [17]. *E. coli* can take up 12 mmol glucose/g dry cell weight (DCW)/h (BNID 109686) [18]. Such uptake could yield a current density of 1 mA/cm^2^, over two orders of magnitude greater than the observed 7.4 × 10^−3^ mA/cm^2^, assuming a similar cellular attachment density (calculation 4).

### Current density at maximum glucose uptake (BNIDs 109686 and 109352)

4 $$\begin{aligned} \left( {12\frac{{\text{mmol glucose}}}{{{{\text{g DCW}}} \times {\text{h}}}}} \right)\left( {\frac{{{\text{g}}\,{\text{DCW}}}}{{10^{3} \,{\text{mg}}\,{\text{DCW}}}}} \right)\left( {\frac{{2\,{\text{mg}}\,{\text{DCW}}}}{\text{mg protein}}} \right) \times \left( {\frac{{0.086\,{\text{mg}}\,{\text{protein}}}}{{{\text{cm}}^{ 2} }}} \right)\left( {\frac{{1\,{\text{h}}}}{{60^{2} \,{\text{s}}}}} \right)\left( {\frac{{24\,{\text{mmol}}\,e^{ - } }}{{1\,{\text{mmol}}\,{\text{glucose}}}}} \right) \hfill \\ \times \left( {\frac{{9.65 \times 10^{4} \,{\text{mC}}}}{{1\,{\text{mmol}}\,e^{ - } }}} \right)\left( {\frac{\text{C}}{{10^{3} \,{\text{mC}}}}} \right) \approx 1\frac{{\text{mA}}}{{{{\text{cm}}}^{ 2} }} \hfill \\ \end{aligned}$$

Clearly, future improvements to this system should focus on increasing metabolic flux. In a related study, Nocera and colleagues showed how rough estimates can be used to improve the design of bioelectrochemical cells for fuel production from sunlight. Here, increased bacterial viability and a redesigned apparatus were offered by the authors as future system improvements [19]. Indeed, in a recent paper, the authors show how a redesigned electrode catalyst increases bacterial viability and improves efficiency of biofuel production by over 20-fold [20]. Together, these examples reveal how rough estimates of cell metabolism and physiology provide important insight into improving a bioprocess.

## Case study 1: evaluation of the capacity of a protein secretion system

Many bioprocesses take advantage of existing, natural biological functions that are engineered with a top-down approach to improve function in the bioreactor environment. For such systems, estimates evaluate native function and guide experiments to modify the system for improved performance. In the following example, we describe how a protein secretion apparatus might be modified for increased protein production. Our targets are difficult-to-produce heterologous proteins. Engineering bacteria to secrete the protein product to the extracellular space is expected to improve production of these toxic or hard-to-purify proteins [21]. To achieve this activity, we adapted the type III secretion system of *Salmonella enterica*. When we started working on this problem, we used estimates to answer three key questions:

1. Can secretion improve the production of proteins with toxic effects?
2. What is the native capacity of the secretion system?
3. If the native capacity is below the desired production level, how should the secretion system be manipulated to achieve increased protein yield?

First, we predict the steady-state intracellular concentration of the toxic protein of interest. Cellular fitness may be increased if the rate of protein secretion is matched with the rate of protein production, such that a low intracellular concentration of the toxic protein is maintained at steady-state, while the toxic protein accumulates in the extracellular space. As an example, we consider that a 50 kDa protein of interest is produced by the ribosomes at a rate of up to 10^3^ proteins/s/cell (calculation 5).

### Maximal translation rate per cell (BNIDs 100059 and 101441)

5 $$\left( {10^{1} \frac{{{\text{amino}}\,{\text{acids}}}}{{{\text{s}} \times {\text{ribosome}}}}} \right)\left( {\frac{{ 1 {\text{ protein}}}}{{ 4 5 0 {\text{ amino}}\,{\text{acids}}}}} \right) \times \left( {10^{4} - 10^{5} \frac{{\text{ribosomes}}}{{\text{cell}}}} \right) \approx 10^{2} {-} 10^{3} \frac{{\text{proteins}}}{{{{\text{s}}} \times {\text{cell}}}}$$

**Fig. 2:**
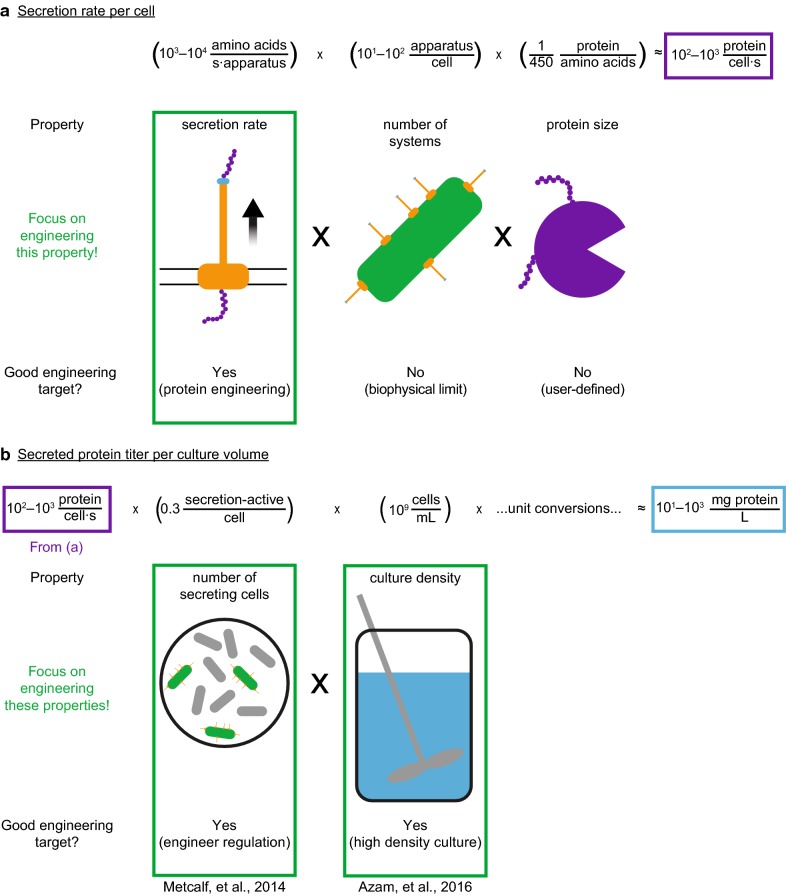
Diagram of estimates used to predict performance of a bacterial protein secretion system. **a** Estimate of per cell protein secretion rate. **b** Estimate of secreted protein titer

Note that in this example, we assume that all ribosomes are actively translating the protein of interest. We also estimate the secretion rate per cell from the type III secretion system using known parameters [1, 22]. Proteins are secreted at a rate of 10^3^–10^4^ amino acids per second per apparatus [23, 24], and each cell has 10^1^–10^2^ secretion apparatus per cell [25]. Therefore we estimate a secretion rate of 10^1^–10^3^ proteins per second per cell for a 50 kDa protein (calculation 6) (Fig. 2a).

### Maximal secretion rate per cell

6 $$\left( {10^{3} - 10^{4} \frac{{{\text{amino}}\,{\text{acids}}}}{{{\text{s}} \times {\text{apparatus}}}}} \right)\left( {10^{1} - 10^{2} \frac{{\text{apparatus}}}{{\text{cell}}}} \right) \times \left( {\frac{{1\,{\text{protein}}}}{{450\,{\text{amino}}\,{\text{acids}}}}} \right) \approx 10^{1} - 10^{3} \frac{{\text{proteins}}}{{{{\text{s}}} \times {\text{cell}}}}$$

Our estimate of the maximal secretion rate is on the same order of magnitude as the maximal translation rate. This suggests that a low intracellular concentration of protein can be maintained by controlling the rate of translation to match the rate of secretion [26]. Thus, we expect that increased production of a toxic protein can be achieved by mitigating cytotoxic effects through maintaining a low steady-state intracellular concentration.

Now we address the second question: What is the capacity of the native protein secretion system? We desire a product titer of 10 g/L in a 72 h batch in order to compete with current industry performance [27, 28]. We estimate the secreted titer by integrating the estimated secretion rate per cell across all cells in the culture. Cultures reach an optical density of ~ 1 OD, equivalent to about 10^9^ cells/mL (BNID 104831) [29]. Only 30% of cells secrete product in this environment [30, 31], such that the predicted secreted protein titer of a 50 kDa protein is 10^1^–10^3^ mg/L in an 8 h batch (calculation 7) (Fig. 2b).

### Native production capacity (BNID 104831)

7 $$\begin{aligned} \left( {8\,{\text{h}}} \right)\left( {\frac{{60^{2} \,{\text{s}}}}{{1\,{\text{h}}}}} \right)\left( {10^{3} - 10^{4} \frac{{{\text{amino}}\,{\text{acids}}}}{{{\text{s}} \times {\text{apparatus}}}}} \right)\times \left( {10^{1} - 10^{2} \frac{{\text{apparatus}}}{{\text{cell}}}} \right)\left( {10^{9} \frac{{\text{cells}}}{{\text{mL}}}} \right) \hfill \\ \times \left( {\frac{{ 0. 3 {\text{ secretion - active cells}}}}{{ 1 {\text{ total cells}}}}} \right)\left( {\frac{{1\,{\text{protein}}}}{{450\,{\text{amino}}\,{\text{acids}}}}} \right) \times \left( {\frac{{1\,{\text{mole}}\,{\text{protein}}}}{{6.02 \times 10^{23} \,{\text{proteins}}}}} \right)\left( {\frac{{50 \times 10^{3} \,{\text{g}}\,{\text{protein}}}}{{1\,{\text{mole}}\;{\text{protein}}}}} \right) \hfill \\ \times \left( {\frac{{10^{3} \,{\text{mL}}}}{{1\,{\text{L}}}}} \right)\left( {\frac{{10^{3} \,{\text{mg}}}}{{1\,{\text{g}}}}} \right) \approx 10^{1} - 10^{3} \frac{{\text{mg}}}{{\text{L}}} \hfill \\ \end{aligned}$$

This range of values agrees well with published titers of ~ 10 mg/L from an 8 h batch [32], supporting the validity of our analysis and suggesting that some of the parameters used in our analysis may be overestimated. This estimate also corresponds to a titer of 10^−1 ^– 10^1^ g/L in a 72 h batch. This analysis reveals that our engineering goal of 10 g/L secreted protein might be achieved by optimizing the native secretion capacity of the type III secretion system, and identifies five parameters that contribute to secreted protein titer:

1. Fraction of cells that are secretion-active.
2. Culture density.
3. Number of apparatus per cell.
4. Secretion rate.
5. Culture time with which proteins are secreted.

This list helps us address our third question—we can now identify parameters to manipulate to achieve the target titer of 10 g/L. An increase in any of the five parameters will result in a proportional change in the product titer. Some parameters, such as culture density [33] or the fraction of cells that are secretion-active [30], can be easily manipulated. Improving the culturing conditions for high cell density culture, while maintaining secretion activity, will cause a concomitant increase in secreted protein titer. Further, the secretion activity on a per cell basis can be manipulated using transcriptional control to increase expression of type III secretion system genes [23]. Other parameters are harder to manipulate experimentally due to physiological limits. For example, if we approximate a cross-sectional area of 1000 nm^2^/apparatus [25], the average *S. enterica* cell (6 µm^2^, BNID 103711) experiences 0.1–1% of the inner membrane surface area occupied by type III secretion system apparatus (calculation 8).

### Surface area occupied by secretion system apparatus (BNID 103711)

8 $$\frac{{{\text{surface}}\,{\text{ area}}\,{\text{ of}}\,{\text{ apparatus} }}}{{\text{cellular}}\,{\text{surface}}\,{\text{area}}} = \left( {1000\frac{{{\text{nm}}^{{\text{2}}} }}{{{\text{apparatus}}}}}\right) \left( {\frac{{{\text{1}}\, {\upmu}{\text{m}}^{{\text{2}}} }}{{(10^{3} )^{2}\,{\text{nm}}^{2} }}} \right) \left( {10 - 100\frac{{{{\text{apparatus}}}}}{{{{\text{cell}}}}}} \right) = 0.1-1\%$$

If the number of apparatus were increased to 10^3^ per cell, this would increase the fraction of the inner membrane occupied by the type III secretion apparatus to 10%, likely decreasing cell viability, as this is a very high fraction of the membrane to devote to a large structure that spans both the inner and outer membrane. Thus, attempting to manipulate this variable would not likely be fruitful in achieving the desired process goal.

In our work, we controlled expression of the secretion system to increase the fraction of cells that are secretion-active by ~ threefold and enable a ~ threefold increase in culture density. By introducing transcriptional control, we manipulate these two key variables simultaneously and achieve a ~ tenfold increase in secreted protein titer [34]. Engineering improvements that were identified by rough estimates resulted in a bacterial strain that was able to produce and secrete heterologous proteins at high titer and enabled the production of difficult-to-express repetitive proteins [35]. We expect increased product titer by further manipulation of the five aforementioned variables. With a goal of 10 g/L in 72 h, we expect that a fivefold further increase in culture density will achieve the target titer of secreted proteins.

## Case study 2: feasibility of enzyme pathway compartmentalization

Estimates can also be used to understand the physical limits of cellular properties and thus establish the upper limit on production of a desired product. Here, we consider the design of subcellular nanoreactors based on naturally occurring organelles. Subcellular structures, such as the carboxysome and the mitochondrion, are spatially and chemically segregated from the rest of the cell to create a specialized metabolic environment [36]. Inspired by these examples from Nature, a subcellular compartment optimized for production of a desired molecule could increase titer, as bioproduction and metabolic homeostasis are decoupled through spatial separation. Towards this goal, our group has sought to repurpose the native bacterial microcompartment (MCP) complex of *S. enterica* for metabolic engineering of diverse bioproducts. When we began this project, we first asked, “Can MCPs be used in the production of an industrially relevant compound at sufficient titer?” Again, we obtain an order-of-magnitude estimate of physical requirements for a desired product yield using rough estimates of the relevant parameters.

**Fig. 3:**
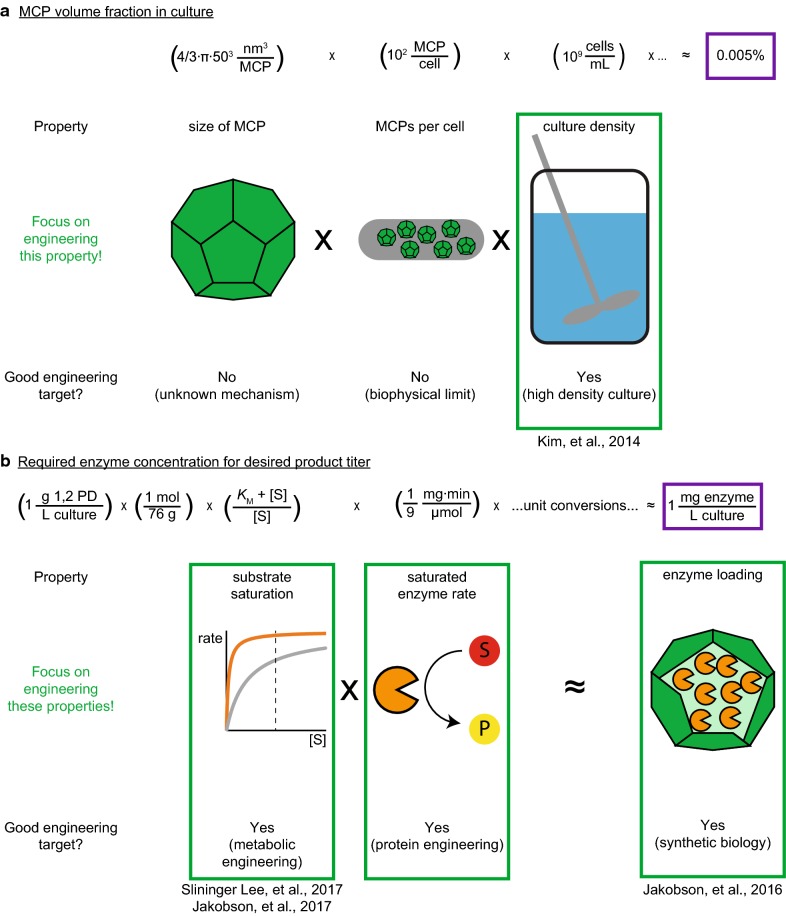
Diagram of estimates used to predict physical requirements to encapsulate a metabolic pathway in bacterial microcompartments. **a** Estimate of fraction of culture volume occupied by microcompartments. **b** Estimate of enzyme concentration required for a desired product yield

We calculate the feasibility of a desired titer from the amount of enzyme that can physically fit within the MCP compartment volume. We note that the MCP is approximately spherical, with a diameter of ~ 100 nm, and that the maximum number of MCPs per cell is likely to be around 100 [37, 38], such that up to 1–10% of the cell volume is occupied by MCPs. At a culture density of 1 OD, MCPs represent 0.005% of the culture volume (calculation 9) (Fig. 3a).

### Culture volume fraction of microcompartments (BNID 104831)

9 $$\left( {\frac{{\frac{4}{3}\pi \left( {50\,{\text{nm}}} \right)^{3} }}{\text{MCP}}} \right)\left( {100\frac{{\text{MCP}}}{{\text{cell}}}} \right)\left( {\frac{{10^{9} \,{\text{cells}}}}{\text{mL}}} \right)\left( {\frac{{\,{\text{mL}}}}{{\left( {10^{7} } \right)^{3} \,{\text{nm}}^{3} }}} \right) \times 100 \approx 0.005\%$$

From this calculation, it is clear that the fractional volume of MCPs in a culture is most significantly determined by the culture density. Does the estimated MCP volume afford enough space for enzymes inside the MCP to produce industrially relevant amounts of a compound of interest? Commodity chemicals are typically produced at concentrations of 50–150 g/L after 48 h of fermentation, and desired titers are dictated by process economics [39, 40]. For this estimate, we set a target of 50 g/L in 48 h of the commodity product 1,2-propanediol (1,2-PD). We calculate the quantity needed of the enzyme with the lowest *k*_*cat*_ from the 1,2-propanediol production pathway, GldA (Table 2), under substrate saturating conditions to see if this target is physically possible. The specific activity of GldA has been experimentally determined to be 5 μmol 1,2-PD/min/mg GldA at saturation [41]. Assuming saturation of GldA, we calculate the minimum concentration of GldA required to achieve this product titer is 50 mg/L (calculation 10) (Fig. 3b).

**Table 2:** Turnover number (*k*_*cat*_) for enzymes in the 1,2-propanediol pathway [41]

Enzyme	*k*_*cat*_ (s^−1^)	Reference(s)
MgsA	220	[42]
AKR	30	[43]
GldA	0.4^a^	[41]

*NR* not reported

^a^Estimated from specific activity

### Concentration of rate-determining enzyme to achieve desired product titer

10 $$\left( {50\frac{{{\text{g}}\;1,2 - {\text{PD}}}}{{{\text{L culture}}}}} \right) \left( {\frac{{1\,\upmu {\text{mol }}1,2 - {\text{PD}}}}{{76\,\upmu {\text{g}}\,1,2 - {\text{PD}}}}} \right) \left( {\frac{{\min \cdot {\text{mg GldA}}}}{{5\,\upmu {\text{mol 1}},2 - {\text{PD}}}}} \right) \left( {\frac{{10^{6} \,\upmu {\text{g}}}}{{1\,{\text{g}}}}} \right) \left( {\frac{{1{\text{h}}}}{{60\min }}} \right) \left( {\frac{1}{{48\,{\text{h}}}}} \right) = 50\frac{{{\text{mg GldA}}}}{{{\text{L culture}}}}$$

Does this concentration of enzyme physically fit inside the MCPs? The approximate density of the GldA enzyme was calculated from the amino acid sequence using the Northwestern peptide properties calculator [44]. The volume of the GldA protein molecule is 5 × 10^4^ Å^3^ and the molecular weight is 40 × 10^3^ g/mol, giving a density of 1.4 g/cm^3^. We then calculate the volume fraction of GldA in MCPs required for our desired product titer (calculation 11).

### Fraction of MCP required for GldA

11 $$\frac{{\left( {\frac{{50\,{\text{mg}}\,{\text{enzyme}}}}{{{\text{L}}\,{\text{culture}}}}} \right)\left( {\frac{{{\text{cm}}^{ 3} \,{\text{enzyme}}}}{{ 1. 4\,{\text{g}}\,{\text{enzyme}}}}} \right)\left( {\frac{\text{g}}{{10^{3} \,{\text{mg}}}}} \right)\left( {\frac{\text{L}}{{10^{3} \,{\text{cm}}^{ 3} }}} \right)}}{{\frac{{0.00005\,{\text{L}}\,{\text{MCP}}}}{{{\text{L}}\,{\text{culture}}}}}} \approx 0.7\frac{{{\text{L}}\,{\text{enzyme}}}}{{{\text{L}}\,{\text{MCP}}}}$$

To produce the desired titer, 70% of the MCP volume must be occupied by GldA. While this fractional loading is high, is shows that the process is feasible and that modest improvements to the process would improve titer. For example, if the cell density was increased to 10 OD, only 7% of the MCP volume would need to be occupied by GldA. This fractional loading of the rate-determining enzyme suggests that MCPs are large enough to fit multiple enzymes in a pathway. The rest of the MCP volume is available for other enzymes in the pathway, as well as metabolites.

The variables we found to affect this calculation are the:

1. Size of MCPs per cell.
2. Number of MCPs per cell.
3. Saturation of the encapsulated enzymes by cognate substrates.
4. Loading of enzymes within the MCP.
5. Maximum reaction rates of the loaded enzymes.
6. Culture density.

Of these variables, the saturation of encapsulated enzymes, loading of enzymes within the shell, and improved culture density all make attractive targets. For the 1,2-PD example, we assumed saturation of the enzyme and an enzyme loading of 70%. Saturation is a feature that depends on culture conditions and shell permeability, as well as on the kinetic constants of the pathway enzymes, and the impact of engineering enzyme turnover and saturation will vary depending on the system [45]. Further, if we consider enzyme engineering to increase the specific activity, we would expect a concomitant increase in product titer, though this is often not trivial. Thus, to increase product titer tenfold, we predict that increasing culture density to 10 OD would suffice. Changing the size or number of MCPs is a much more challenging target, as the mechanisms controlling these phenotypes are unknown, and would not improve titers by orders of magnitude.

Therefore, in our work we first set out to improve control over MCP expression, the permeability of the MCP shell, and enzyme loading. Controlling expression of MCP genes enables increased culture density [46]. Further, controlling permeability of the protein shell to metabolites changes the concentration of substrates in the MCP [45, 47], enabling operation at substrate-saturating regimes. Finally, the loading of enzymes in the MCP can be modulated via the targeting sequence and expression levels [48], and the low fractional volume of enzymes in MCPs enables modulation of enzyme loading. If further improvements are needed, increasing the specific activity of the enzyme—by engineering or identifying more active homologs—might be the next best target because it could improve yield by an additional one or more orders of magnitude.

## Using rough estimates in new bioprocesses

**Fig. 4:**
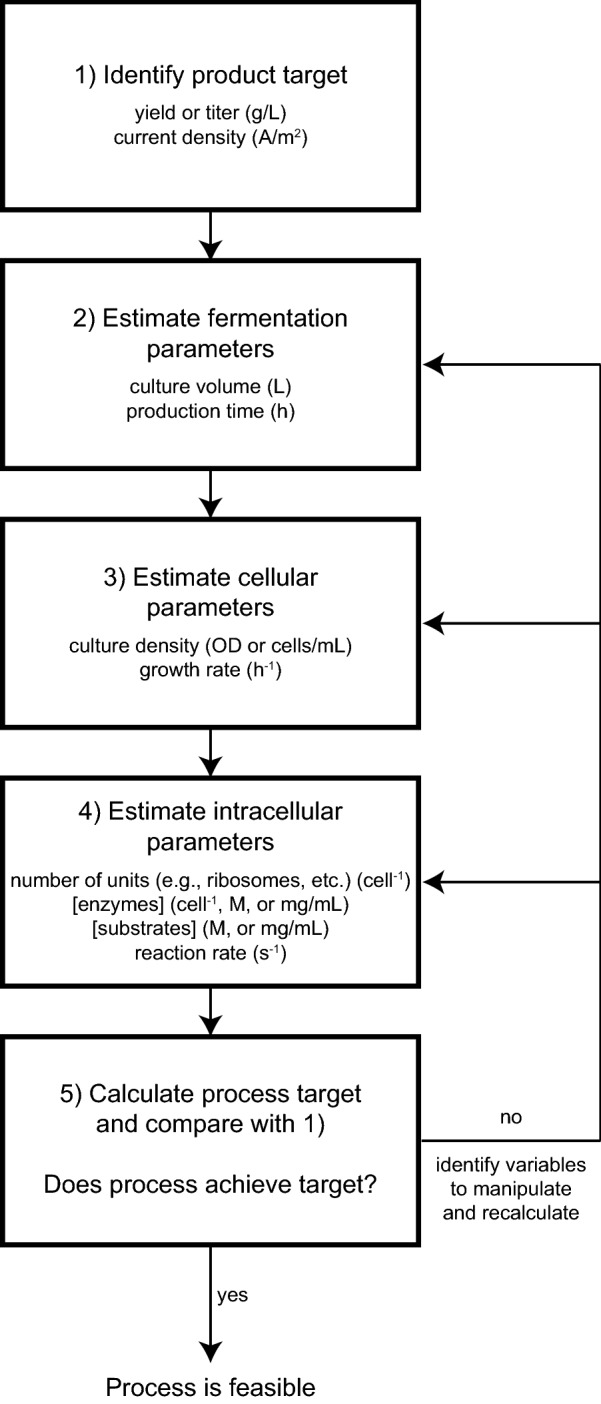
Decision scheme for making rough estimates of process feasibility and variables to target for optimization. Estimates come from primary research, the BioNumbers database (http://bionumbers.hms.harvard.edu), and intuition

The above examples highlight the value of the rough estimates in successful bioprocess engineering projects. At the project outset, estimates help to determine if the process is feasible. Further along in the project, estimates identify properties to improve. In all cases, successful use of estimates will require a keen biological intuition. To help build an intuition, we encourage others working in this area to use a decision scheme (Fig. 4) to guide their process analysis and engineering efforts, and emphasize that moving through this scheme requires balancing the potential payoff with the amount of effort it will require. Moreover, we expect that the majority of successful projects used such a decision scheme, yet few have discussed their rough estimates in the literature alongside the project results. Given its importance, we strongly promote the inclusion of rough estimates in all future published work in the synthetic biology field. It will benefit new researchers learning to analyze new processes, as well as everyone interested in understanding the context of the work, including why certain parameters were not chosen for optimization.

## Abbreviations

BNID: BioNumber identification number
DCW: dry cell weight
MCP: bacterial microcompartment
